# Porous AlGaN-Based Ultraviolet Distributed Bragg Reflectors

**DOI:** 10.3390/ma11091487

**Published:** 2018-08-21

**Authors:** Peter Griffin, Tongtong Zhu, Rachel Oliver

**Affiliations:** Department of Materials Science and Metallurgy, University of Cambridge, 27 Charles Babbage Road, Cambridge CB3 0FS, UK; phg23@cam.ac.uk (P.G.); rao28@cam.ac.uk (R.O.)

**Keywords:** nitride, porous, electrochemistry

## Abstract

Utilising dislocation-related vertical etching channels in gallium nitride, we have previously demonstrated a simple electrochemical etching (ECE) process that can create layered porous GaN structures to form distributed Bragg reflectors for visible light at wafer scale. Here, we apply the same ECE process to realise AlGaN-based ultraviolet distributed Bragg reflectors (DBRs). These are of interest because they could provide a pathway to non-absorbing UV reflectors to enhance the performance of UV LEDs, which currently have extremely low efficiency. We have demonstrated porous AlGaN-based UV DBRs with a peak reflectance of 89% at 324 nm. The uniformity of these devices is currently low, as the as-grown material has a high density of V-pits and these alter the etching process. However, our results indicate that if the material growth is optimised, the ECE process will be useful for the fabrication of UV reflectors.

## 1. Introduction

Deep Ultraviolet (DUV) light is crucial for a wide range of industrial applications from curing processes to sterilisation. This light is predominantly produced by mercury (Hg) lamps, despite their limited lifetimes, use of high voltages, and the requirement for highly toxic Hg. AlGaN with a high Al content emits light deep into the UV and AlGaN-based LEDs therefore presenting a candidate for a UV light source, which could mitigate all of these disadvantages. Additionally, DUV laser diodes have broad potential applications for high precision laser scribing and data storage [1]. Unfortunately, AlGaN optoelectronic devices operating in the DUV currently exhibit very low efficiency. This is due to various factors that are associated with increasing aluminium content of the AlGaN material required to reach shorter wavelengths [2]. Briefly, these problems are due to: low internal quantum efficiency due to high defect density [2]; high light absorption from p-GaN layers [3] necessary to provide effective hole injection; and, the strong anisotropic emission of AlGaN, which causes c-plane grown AlGaN to preferentially emit laterally [4].

Distributed Bragg reflectors (DBRs) are structures that are made from alternating layers of different refractive index. They can be highly reflective and the stopband of the reflection peak is tuneable by varying the optical thickness of the constituent layers. They offer opportunities in AlGaN light sources both to create cavities to form vertical cavity surface emitting lasers, as well as boosting the external quantum efficiency of DUV LEDs. In the nitrides, conventional DBRs are made by growing alternate layers of material, which have differing refractive index. The thickness of the layers is designed such that interference resulting from multiple reflections produces a mirror whose reflectivity is high at a certain wavelength. The reflectivity of epitaxial DBRs is limited by the low refractive index contrast between nitride materials requiring high numbers of DBR pairs, whilst a high number of strained layers causes high dislocation densities and longer growth times. For example, AlN and AlGaN have a maximum refractive index contrast in the range 7–11%. An AlN/AlGaN DBR structure has achieved 97% reflectivity down to 220–250 nm, but required 30.5 pairs [5].

Alternatively, a much higher refractive index contrast can be achieved with layers of the same lattice constant by creating porosity in doped layers. In the visible spectrum, we have previously demonstrated highly reflective wafer-scale DBRs that are made from alternating layers of GaN and porous GaN [6]. This process uses an electrochemical etching (ECE) process to create pores in subsurface doped GaN without significant damage to the non-intentionally doped (NID) GaN layers. Porous AlGaN DBRs with 90% reflectivity at 379.3 nm have been demonstrated to enhance the photoluminescence of LEDs [7], and they have significantly enhanced the electroluminescence of GaN LEDs around 360 nm [8]. Both of these demonstrations required laser scribed access trenches to be patterned in order to create subsurface porosity, which increases the complexity and the cost of device production.

In this paper, we apply the wafer-scale porosification method that we developed for GaN DBRs [6] to AlGaN structures and explore the material issues that affect the realisation of porous UV DBRs. This presents a potential route to forming highly reflective single alloy porous DBRs across whole wafers in a single process step.

## 2. Materials and Methods

**Figure 1:**
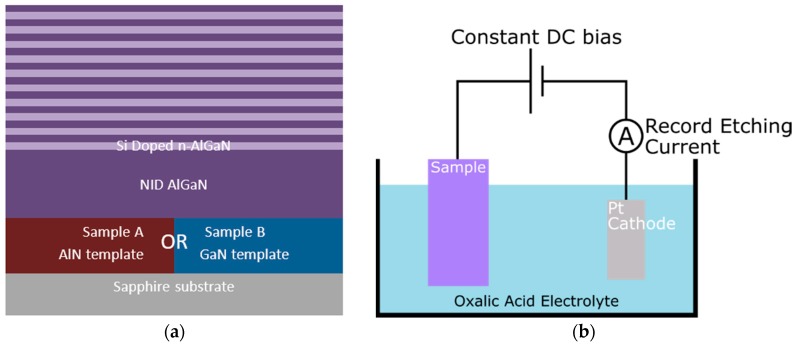
(**a**) Schematic diagram of the AlGaN samples grown on two separate templates, sample A on AlN and sample B on GaN. The distributed Bragg reflectors (DBRs) are made of ten pairs of ca. 50 nm of Si doped (1 × 10^19^ cm^−3^) n-AlGaN and ca. 35 nm of non-intentionally doped (NID) AlGaN. (**b**) Experimental setup of the electrochemical etching (ECE), as described in [6].

All of the samples were grown by metal-organic vapour phase epitaxy in a 6 × 2 inch Thomas Swan close-coupled showerhead reactor on c-plane sapphire substrates while using trimethylgallium, trimethylaluminium, and ammonia as precursors, hydrogen as a carrier gas, and silane for n-type doping. The AlGaN structures were grown with an aluminium alloy fraction around 60% and consisted of ten alternating pairs of Si doped n-AlGaN of ca. 50 nm with a nominal doping density of 1 × 10^19^ cm^−3^ and NID AlGaN layers of ca. 35 nm, as shown in Figure 1a. The topmost layer was NID AlGaN. The structures were grown concurrently on two templates to compare the influence of defects and of strain state on pore formation. Sample A used a NID AlN template meaning that the AlGaN was grown in compression and sample B used a NID GaN template meaning that the AlGaN was grown in tension.

ECE experiments were conducted in a two-electrode cell at room temperature, without UV illumination, following the method presented in [6] and shown in Figure 1b. The AlGaN sample formed the anode and a platinum plate the cathode with a 0.25 M Oxalic acid electrolyte. The anodisation process was carried out in a constant voltage mode at 8 volts controlled and monitored by a Solartron 1287A potentiostat (Ametek, Oak Ridge, TN, USA). After anodisation, samples were rinsed with deionized water and blow dried in a stream of air. An anodization voltage of 8 V was chosen following a series of experiments on doped single layer AlGaN structures with voltages between 6 V and 12 V. 8 V produced a sub-surface porous structure in a reasonable time frame without significant damage to the NID layers, which was seen at higher voltages.

Secondary electron scanning electron microscope (SEM) images were obtained while using either an FEI XL30 (FEI, Eindhoven, Netherlands) or a Nova NanoSEM (FEI, Hillsboro, OR, USA). All of the images show a cross-section of the layered structure cleaved after etching, such that they are indicative of the inside structure of the sample away from the edge. The surface morphology data were obtained using peak force tapping mode on a Veeco Dimension Pro atomic force microscope (AFM, Veeco, New York, NY, USA) with a Scansyst-air-HR tip.

Estimating porosity from SEM data was done while using ImageJ analysis software (version 1.8.0_66). The image was first despeckled and then a porous region was cropped, taking care to include only the porous layer. This section was binarized while using the phansalker method of local threshold, as described in [9]. This takes account of locally varying differences in intensity and it was found to give the best results from a variety of thresholding methods. The “analyze particles tool” was then used to find the total area of the dark regions in this box, which should correspond to pores. The radius of the local area that is used to threshold each pixel is a key parameter. A radius of five pixels was found to give a binary image that best represented the SEM data.

To obtain reflectivity spectra, an Ocean Optics system (Ocean Optics, Largo, FL, USA) was used, which consisted of a reflection probe the USB4000-UV-VIS spectrometer and a DH-2000 UV-Vis light source illuminating the sample from 190–2500 nm. Reflectivity was calibrated with a polished aluminium reference standard (Ocean Optics Stan-SSH) [10].

## 3. Results

**Figure 2:**
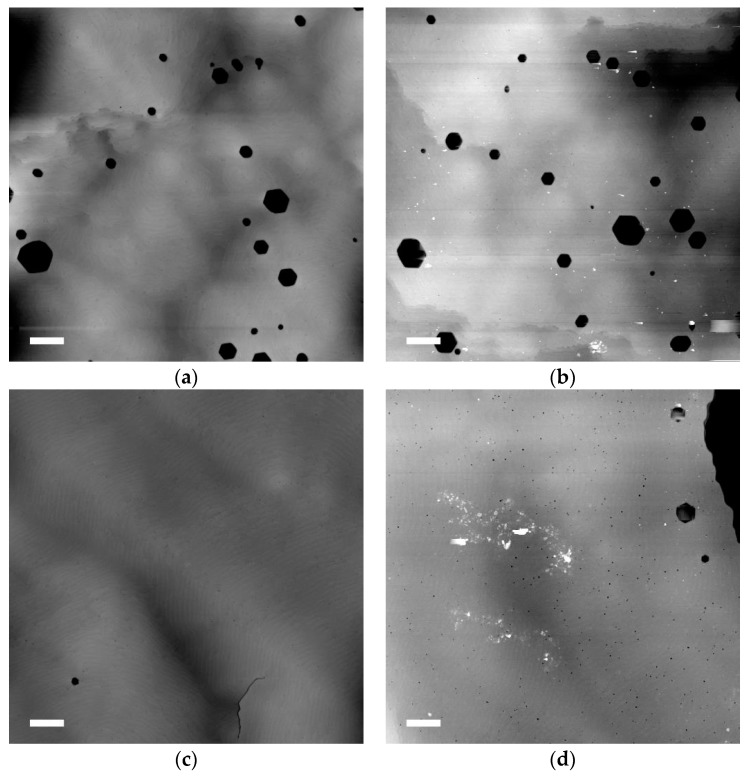
Atomic force microscope (AFM) images with a 5 µm scan size for: (**a**) sample A as grown; (**b**) sample A after ECE; (**c**) sample B as grown; and, (**d**) sample B after ECE. Lateral scale bars are 500 nm and the z range is 20 nm.

Figure 2 shows AFM images of the surface of sample A, before (a) and after (b) ECE. Both of these images show large hexagonal surface pits with a density ca. 7 × 10^7^ cm^−2^ and diameters in the range 50–500 nm, which suggests that the etching process does not change the surface morphology. AFM images of the surface of sample B are also in Figure 2, before (c) and after (d) ECE. Figure 2c shows one hexagonal V-pit measuring around 80 nm across and a fissure around 1 µm long. The surface is also pockmarked with a very high density of dislocation pits that measure a few tens of nm across and only around 1 nm deep. Figure 2d also shows dislocation pits, which appear slightly larger, some as large as 50 nm across and a depth of 10 nm. This difference is very small and it may be caused by a difference in AFM probe quality, rather than any real change in the surface. The etched sample also shows three larger V-pits of a few 100 nm across and some white marks that are caused by dirt. Optical data (not shown here) revealed that sample B also has a network of cracks across the entire surface of the as-grow material, as expected for an AlGaN layer under tension on a GaN template.

**Figure 3:**
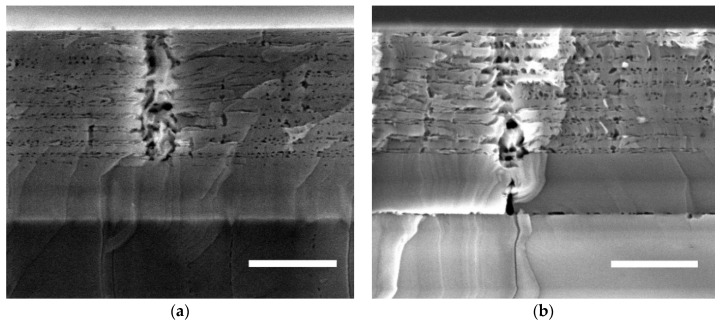
Cross-sectional scanning electron microscope (SEM) images of the porous structure of the AlGaN DBR. (**a**) Sample A, grown on an AlN template; and, (**b**) sample B grown on a GaN template. Scale bars are 500 nm.

Figure 3 shows cross-sectional SEM images of samples A and B after etching. The ECE process has created pores in all ten layers of n-AlGaN in both samples. There are many sites that show large, disordered channels between the layers, with porosity forming in the NID AlGaN layers as well as the doped material. It is difficult to accurately measure the porosity of the porous layers, since it varies substantially, even within the small area illustrated. A rough estimate was made while using the method described above that the layers are around 20% porous, which is significantly lower than the porosity observed in GaN layers etched under similar conditions.

**Figure 4:**
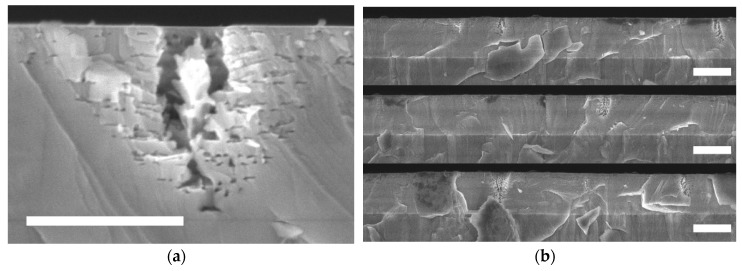
SEM micrographs of sample A, the AlGaN DBR on an AlN template, following a short etch. (**a**) shows a porous column with a scale bar of 500 nm. (**b**) shows 30 µm of continuous material in three images, showing the distribution of the columns. Scale bars are 1 µm.

Figure 4 contains cross-sectional SEM images of sample A after a short etch of 4000 s, at which point only the first few layers are fully porous. Figure 4a shows a porous channel that has a 180 nm wide opening to the surface. The material next to the channel has become porous 7 or 8 layers into the doped/undoped stack, whilst further from the channels material in the lower layers has not yet been porosified. The images of Figure 4b make up 30 µm of continuous SEM imaging, which allows for the density of the porous columns to be calculated. There are seven columns within the 30 µm, as well as two porous regions that may correspond to a nearby column out of the plane of the cleave.

**Figure 5:**
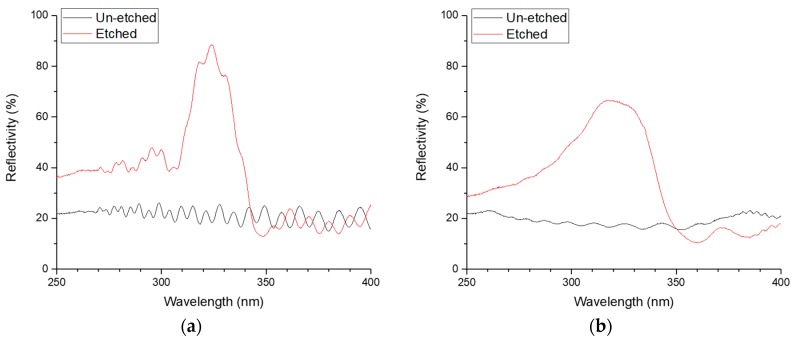
Reflectivity measurements of the two AlGaN DBRs (**a**) sample A and (**b**) sample B.

Measurements of optical reflectivity were made on both sample A and sample B, and they are shown in Figure 5. The etching process creates a peak in reflectivity at around 320 nm in both samples. Sample A has a peak reflectivity of 89% centred at 324 nm with a full-width at half maximum (FWHM) of 30 nm. The reflectivity of the un-etched sample stays at around 22% across all wavelengths. Sample B is less reflective with a peak reflectivity of 67% measured at 318 nm and a wider FWHM of 67 nm, skewed towards shorter wavelengths. The reflectivity of the un-etched sample is around 17%. The system that is used to make the measurements has a spot size of several mm, so, the measurements are macroscopic.

## 4. Discussion

Figure 3 shows that the ECE process has fully porosified all of the doped layers in both samples, but the pore morphology is highly non-uniform. There are frequent and large channels of porosity that pass through the NID layers, which disrupt the layer structure. The SEM data of the short-etched sample in Figure 4 shows that these channels etch early on in the process, which suggests that they correspond to pits that are open to the electrolyte. The diameter of the pit opening in the top image of Figure 4a is 180 nm, which is within the range of V-pit size seen in the AFM data of Figure 2a of 50–500 nm. The size of the other column openings in Figure 4b are also in this range and there is no evidence in this SEM data of other surface features that might relate to the V-pits. Equally, there is no evidence in the AFM data post-etching that additional surface structures have formed, which could correspond to the channels that are seen in SEM. We can therefore conclude that the V-pits seen in the AFM data are responsible for the channels that are seen in the SEM that disrupt the porous structure.

The large pits we observe are similar to previously reported hexagonal V-pits that form in AlGaN grown on AlN templates on sapphire [11]. One model for V-pit formation is that growth is slowed by the segregation of impurities around a threading dislocation and the slower growth forms a pit. These pits expose semi-polar crystal planes and the growth continues on these planes, which grow slower than the surface c-plane. The slower growth of these planes causes the pit to widen as growth continues and the dominant exposed planes create a distinctive hexagonal shape. Reducing the density of threading dislocations in the template may reduce the pit density, although the density of large pits is much smaller than the dislocation density in the templates in either sample A (AlN template for which the dislocation density is >10^9^ cm^−2^) or sample B (GaN template for which the dislocation density is ca. 3 × 10^8^ cm^−2^). Changes to the growth conditions to alter the energy and/or growth rate of the semi-polar facets are likely to be necessary to eliminate these pits. The formation of semi-polar facets during growth is known to increase the incorporation of impurities including oxygen leading to unintentional n-type doping. This may explain the etching of the nominally undoped layers in the V-pit region and the formation of locally disordered structure [12]. In our previous work on GaN DBRs [6], we observed high uniformity in the porous layers and negligible damage to the NID layers, but crucially those samples do not exhibit large V-pits. In the GaN case vertical etching pathways are due to dislocations. Due to the chaotic structure caused by the channels that are associated with the large V-pits, it is unclear whether smaller channels also form around dislocations without large V-pits in the AlGaN case. V-pit free AlGaN DBRs are needed to verify this.

Based on the data in Figure 3a, an approximate value for porosity away from the disordered channels was calculated at 20%. Taking this as a starting point for the average porosity throughout the 50 nm Si-doped AlGaN layer and assuming that the 35 nm NID AlGaN layer is entirely without pores implies refractive indices for Al_0.6_Ga_0.4_N of 1.98 and 2.23 for the porous and non-porous layers respectively. A simple transfer matrix model (TMM) can then be used to estimate the expected reflectivity of such a structure. This predicts a peak reflectivity of 85% at a wavelength of ca. 350 nm and with a full width half maximum (FWHM) to the stop band of around 50 nm. Comparing this result first to the data from sample A, we see that the peak reflectivity achieved is surprisingly similar to the model value despite the low uniformity of the porous structure. The peak occurs at a slightly shorter wavelength than the TMM would predict, probably due to inaccuracies in the calibration of the growth rates, and hence inaccuracies in the estimated layer thicknesses in the sample. Whilst the match between the model and the data is not perfect, the fact that the peak reflectivity value is similar to the model value implies that it is not the microscopic non-uniformity occurring due to the surface pits, which most limits the experimental reflectivity, but rather the low porosity achieved. The low porosity is probably attributable to the lower conductivity of AlGaN, relative to GaN doped with Si at similar concentrations, which results in a lower applied potential at the sample/electrolyte interface.

When comparing the data from sample A and sample B: sample A is both more reflective and has a narrower FWHM than sample B. Sample B is, however, afflicted with a network of cracks which will certainly impact the uniformity of the reflectivity, not only through their obvious impact on the surface morphology, but also by providing an alternative etch pathway. If cracking occurred after growth during cooling, the cracks then provide a pathway to an exposed edge of the doped/undoped multilayer, so that the etch can access the doped layers and etching can occur laterally, as has been achieved for GaN DBRs in which trenches that are formed by reactive ion etching cut through a doped/undoped multilayer to provide access routes for the electrochemical etch [13]. Hence, the low reflectivity and wide full width half maximum of sample B are likely to be attributable to broad scale variations of the etching process across the surface on a scale that is similar to the crack spacing, which varies across the surface from a few microns to several tens of microns.

## 5. Conclusions

We have demonstrated porous AlGaN-based UV DBRs while using a simple one-step ECE process with a peak reflectance of ca. 89% at 324 nm. The uniformity of the porous structure is poor due to the high density of large surface V-pits present in the as-grown material. These disrupt the vertical etching process and form large chaotic porous regions in the NID AlGaN layers. However, for the most successful sample, the peak reflectivity agrees well with a simple transfer matrix model based on estimated porosity from SEM data, which suggests that it is the fairly low porosity that is limiting the reflectivity rather than the non-uniformity.

This electrochemical approach is a promising method for the realisation of highly reflective UV DBRs for AlGaN-based UV optoelectronics. In considering how to achieve improved UV DBRs while using this approach, it is clear that strain management is paramount. Whilst, if cracking is avoided, V-pit formation does not appear to dominate the reflectivity, the presence of such large V-pits would prohibit the use of such DBRs for further device fabrication. To achieve higher reflectivity for a given number of DBR pairs it will be important not only to optimise the epitaxy but also to explore the ECE parameter space further to increase the porosity of the etched AlGaN layers and hence the refractive index contrast.

## Supplementary Materials

This publication is supported by multiple data sets, which are openly available here: https://doi.org/10.17863/CAM.26245.
